# Reappearance from Obscurity: Mammalian Rad52 in Homologous Recombination

**DOI:** 10.3390/genes7090063

**Published:** 2016-09-14

**Authors:** Kritika Hanamshet, Olga M. Mazina, Alexander V. Mazin

**Affiliations:** Department of Biochemistry and Molecular Biology, Drexel University College of Medicine, Philadelphia, PA 19102, USA; kritika.u.hanamshet@drexel.edu (K.H.); olga.mazin@drexelmed.edu (O.M.M.)

**Keywords:** genetic recombination, DNA double-strand break repair, DNA strand exchange, BRCA1, BRCA2, RAD51, synthetic lethality

## Abstract

Homologous recombination (HR) plays an important role in maintaining genomic integrity. It is responsible for repair of the most harmful DNA lesions, DNA double-strand breaks and inter-strand DNA cross-links. HR function is also essential for proper segregation of homologous chromosomes in meiosis, maintenance of telomeres, and resolving stalled replication forks. Defects in HR often lead to genetic diseases and cancer. Rad52 is one of the key HR proteins, which is evolutionarily conserved from yeast to humans. In yeast, Rad52 is important for most HR events; Rad52 mutations disrupt repair of DNA double-strand breaks and targeted DNA integration. Surprisingly, in mammals, Rad52 knockouts showed no significant DNA repair or recombination phenotype. However, recent work demonstrated that mutations in human RAD52 are synthetically lethal with mutations in several other HR proteins including BRCA1 and BRCA2. These new findings indicate an important backup role for Rad52, which complements the main HR mechanism in mammals. In this review, we focus on the Rad52 activities and functions in HR and the possibility of using human RAD52 as therapeutic target in BRCA1 and BRCA2-deficient familial breast cancer and ovarian cancer.

## 1. Introduction

Homologous recombination (HR) is a highly conserved pathway that plays a major role in repair of double-strand breaks (DSBs), the most harmful type of DNA damage, which are induced by ionizing radiation (IR), chemical agents or during repair of stalled replication forks and incomplete telomere synthesis. HR is also important for faithful segregation of chromosomes in eukaryotes during meiosis. HR is an error-free process because it utilizes an intact homologous DNA sequences as a template for the repair of DSBs unlike alternate DSB repair pathways, non-homologous end joining (NHEJ) or microhomology-dependent end-joining (MHEJ), that are error-prone [1,2]. 

**Figure 1:**
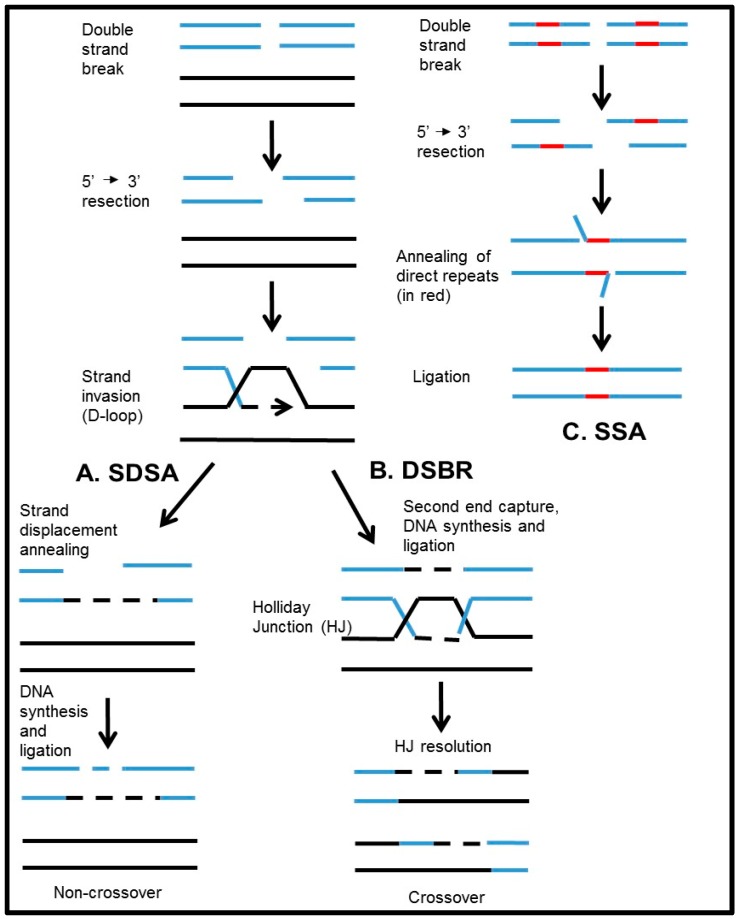
The DNA double-strand break (DSB) repair by Homologous Recombination (HR). The initial steps involve, 5’ to 3’ exonucleolytic processing of DSB ends to produce 3’-ssDNA tails, formation of RAD51-ssDNA filaments, search for homology and strand invasion into the homologous duplex DNA-template leading to the formation of displacement loops (D-loop). Then, HR may proceed either by (**A**) SDSA forming non-crossover products or (**B**) DSBR forming crossover products. When DSB are flanked by direct repeats, the break may be repaired by single strand annealing (SSA) mechanism mediated by repeated DNA sequences (**C**).

The process of HR involves recognition and enzymatic processing of the DSB to produce 3’-ssDNA tails, formation of Rad51-ssDNA filaments that search for homology and promote strand invasion into the homologous duplex DNA-template leading to the formation of a displacement loop (D-loop) intermediate, which provide a template for DNA polymerase to extend the invading DNA strand. After D-loop formation, HR may proceed by two major alternative mechanisms. In the mechanism known as synthesis-dependent strand annealing (SDSA), D-loops dissociate and the extended invading strand re-anneals with the resected second end of DSB forming non-crossover recombinants (Figure 1A). In contrast, in the canonical DNA double-strand break repair mechanism (DSBR), the displaced strand of the D-loop may anneal with the second resected end of the DSB leading to formation of double D-loops that are converted to Holliday junctions. Branch migration and resolution of Holliday junctions lead to formation of crossover recombinants (Figure 1B) [1,3]. While in generative cells crossing over between homologous chromosomes is essential for their accurate segregation, in somatic cells crossing over may lead to excessive loss of heterozygosity. The mechanisms were proposed that channels HR intermediates from DSBR into SDSA mechanism reducing frequency of crossing overs. Thus, Holliday junctions can be dissolved by action of BLM-Topo3α-RMI complex [4]. In addition, double D-loops can be dissociated by Rad54 through its branch migration activity [5].

In some cases, single-end DSBs are formed; for instance, during replication fork collapse. It was proposed that these DSBs are repaired by the mechanism known as break-induced replication (BIR) through strand invasion into a homologous dsDNA followed by replication to the chromosome end. Because BIR results in an extensive loss of heterozygosity, it thought that this mechanism is suppressed when DSBs can be repaired by other more conservative HR mechanisms [6,7].

Rad52 (Radiation sensitive 52), an important HR protein, was initially identified in *S. cerevisiae* during a genetic screen for mutants sensitive to IR [8]. A number of other HR proteins—Rad50, Rad51, Rad54, Rad55, Rad57, Rad59, Mre11, and Xrs2—were also identified during the genetic screening; they belong to the RAD52 epistasis group [8,9]. Among all the members of this group, Rad52 has the strongest effect on HR and DNA repair in *Saccharomyces cerevisiae*. Moreover, rad52 mutants are most IR-sensitive among all *S. cerevisiae* single mutants. Apart from defects in DSB repair, rad52 mutants also show deficiency in mating-type switching [10], meiosis, spore viability [8,10], and homologous DNA integration into genome [11].

Given an important role of Rad52 in yeast, it came as a surprise that Rad52 knockout mouse showed nearly normal DNA repair and HR phenotype [12]. However, recent work from S. Powell’s group demonstrated that Rad52 has an essential role maintaining the viability of mammalian cells, when BRCA1, BRCA2 or several other HR proteins including PALB2 and RAD51 paralogs (RAD51B, C, D, and XRCC2,3) are inactivated or depleted [13,14,15]. These data indicate a complex organization of the HR machinery in mammalian cells and suggest that RAD52 may play a back-up role, when an alternative HR mechanism(s) that depends on BRCAs and several other HR proteins are unavailable. It is to note, that in contrast to many other eukaryotes *S. cerevisiae* genome does not encode *BRCA1* and *BRCA2* homologs suggesting that Rad52 may perform their functions in yeast. These findings also suggest that human RAD52 may present a therapeutic target in hereditary BRCA1/BRCA2/PALB2/RAD51 paralogs-deficient breast cancer and ovarian cancer [16,17,18,19]. 

Despite extensive genetic and biochemical studies, the exact function(s) of Rad52 remains to be elucidated. Furthermore, recent findings have extended the spectrum of possible Rad52 activities in the cell. Thus, a new role of Rad52 in RNA-templated DNA repair has emerged [20]. Here, we review the functions and new therapeutic applications of RAD52 in light of these new discoveries. More comprehensive information on human RAD52 and its yeast ortholog may be found in a number of excellent previous reviews [1,9,14,21,22]. 

## 2. The Role of RAD52 in HR in Mammals

Unlike yeast rad52 mutants which show strong deficiency in nearly all types of HR events including DSB repair, RAD52^−/−^ mice are viable, show only moderate decrease in HR, no DNA damage sensitivity, fertile without abnormalities or cancer predisposition [12]. Similarly, Rad52^−/−^ chicken B-cell line DT40 cells also showed only moderate decrease in targeted integration frequency with no significant DNA damage sensitivity [23]. Still, evidence exists that Rad52 plays a role in HR in mammalian cells. Overexpression of Rad52 in monkey cells increases their resistance to IR, indicating the importance of Rad52 in DSBs repair [24]. In addition, in murine fibroblasts it was found that in response to DNA damage with IR or methylmethanesulfate (MMS) Rad52 fused with green fluorescent protein (GFP) forms nuclear foci that partially overlap with either Rad50 foci or with Rad51 foci [25,26]. These results are consistent with the role of Rad52 in DNA repair in mammalian cells. Since Rad50 and Rad51 foci were shown to not overlap [27], these results, taken together, may indicate two distinct modes of Rad52 action: Rad51-dependent and Rad51-independent. 

Recently, the Powel’s group presented important evidence for the role of RAD52 in HR in human cells. They demonstrated that depletion of human RAD52 is synthetically lethal with mutations in any of several other members of the HR pathways including BRCA1, BRCA2, PALB2, and RAD51 paralogs [13,15,28]. These results, in parallel with the previous findings in chicken DT40 B-cells that Rad52 mutations are synthetically lethal with Xrcc3 mutations [29], indicate that, at least, two alternative HR mechanisms operate in mammalian/vertebrate cells, and that one of them, the Rad52-dependent mechanism, is essential for cell viability in the absence of BRCA1, BRCA2, PALB2, or RAD51 paralogs. However, the thorny question remains which of the Rad52 specific activities are responsible for viability of the cells deficient in BRCAs and several other HR proteins. 

Several Rad52 activities were previously identified. First, Rad52 promotes annealing of complementary ssDNA or ssRNA strands [30,31,32] and DNA strand exchange (DNA pairing) between homologous DNA [33,34]. In addition, yeast Rad52 facilitates loading of Rad51 on ssDNA covered by Replication Protein A (RPA), a ubiquitous ssDNA binding protein [35,36,37]. The latter activity, known as a mediator activity, may seem to be the most relevant to the function of human RAD52 in BRCA2-deficient cells, because it may substitute for the known mediator activity of BRCA2 [38]. However, in contrast to yeast Rad52, the mediator activity was not demonstrated for human RAD52 in biochemical studies [39]. Below we review possible contribution of Rad52 activities to HR in mammalian cells. 

## 3. Activities of Rad52 Protein

### 3.1. ssDNA Annealing Activity

Both yeast and human RAD52 promote annealing of complementary ssDNA strands [30,31,32]. While ssDNA annealing activity is quite common among HR protein, in most cases, e.g., for the members of RECQ family, it is abolished in the presence of RPA [40,41]. In contrast, Rad52 ssDNA annealing occurs in the presence of RPA, which may indicate the biological role of this Rad52 activity in the cell [31]. Two non-exclusive mechanisms were proposed for ssDNA annealing by human RAD52. First, it was suggested based on the structural and biochemical data that RAD52-ssDNA complex, in which the ssDNA bases are displaced outward, interacts with uncoated ssDNA or with ssDNA-RPA complex [42]. Second, it was proposed that ssDNA annealing involves interaction between two or more RAD52-ssDNA complexes [42,43]. In addition, the RAD52 secondary DNA binding site may play a role during ssDNA annealing by binding complementary ssDNA [33,44] (see DNA invasion or strand exchange section below). 

In yeast and mammalian cells, Rad52 ssDNA annealing activity is likely responsible for the Rad51-independent DSB repair pathway through single strand annealing (SSA) between repeated DNA sequences (Figure 1C) [2,45,46]. Like in SDSA or DSBR mechanisms (Figure 1A,B), in SSA the DSBs undergo exonucleolytic resection, but instead of invading homologous DNA templates, the resected ends anneal to each other in a case where DSBs are flanked by fortuitous repeated sequences, e.g., Alu repeats. The SSA is an error-prone process because it results in deletion of DNA sequences between the direct repeats and also one of the repeats [30,32,47,48,49]. Using the chromosomally integrated DSB repair reporters containing repeated sequences of the *GFP* gene, Stark et al., provided evidence that the mammalian Rad52, is involved in repairing DSBs by SSA in an Rad51-independent manner [45]. In contrast to Rad52, Rad51 was shown to suppress SSA, but promote HR through SDSA. When the activity of Rad51 or BRCA2 is impaired, the repair of DSBs is shifted towards SSA indicating interplay between two different HR sub-pathways [45]. However, when BRCA1 was inactivated, it results in a decrease of Rad52 mediated SSA [45], which likely reflects the role of BRCA1 in exonucleolytic DSB end processing that is required for both SDSA and SSA [50] (Figure 1). Still, disruption of BRCA1 has an even stronger inhibitory effect on Rad51-dependent recombination. Overall, based on these data, one may suggest that Rad52 annealing activity contributes to the SSA error-prone mechanism that plays a relatively larger role in BRCA-deficient cells compared with normal cells. However, it is unclear whether this Rad52 activity alone is sufficient to support the viability of BRCA-deficient cells.

Apart from SSA, ssDNA annealing activity of Rad52 was suggested to play a role in the second DNA end capture during Rad51-dependent DSB repair [51]. After the initial strand invasion mediated by Rad51, Rad52 may bind to the resultant displaced ssDNA strand and promote its annealing to the resected second end of DSB, which results in formation of double D-loops and consequently of double Holliday junctions (Figure 1B). In vitro reconstitution studies with purified Rad52 and other HR enzymes support this model [5,51,52]. However, the fact that Rad52-knockout mice are fertile indicates that their meiotic recombination is largely intact and that Rad52 ssDNA annealing activity is not critical for double-Holliday junction formation. Nevertheless, the role Rad52 in the second end capture cannot be excluded; this Rad52 activity may merely be masked by similar activities of other proteins that remain to be identified. BRCA2 apparently does not possess this activity [38]. 

Recently, it was shown that both yeast and human RAD52 can also promote annealing between complementary ssDNA and ssRNA strands [20]. Importantly, this Rad52 activity is not abolished in the presence of RPA, indicating its possible biological role. It was suggested that Rad52-promoted annealing between ssRNA and ssDNA contributes to the Rad52 role in a novel mechanism of RNA-dependent DSB repair [20]. However, the role of this Rad52 activity in viability of BRCA-deficient cells remains to be elucidated [20].

### 3.2. Stimulation of Rad51

Both yeast and human RAD52 stimulate DNA strand exchange activity of their Rad51 counterparts [35,36,37,53], although stimulation of human RAD51 by human RAD52 was reported only for condition when RAD51 is only partially active or present in sub-optimal amounts [53]. Two modes of stimulation were reported: (i) Rad52 may act as a mediator that alleviates an inhibitory effect of RPA during Rad51 filament assembly on ssDNA [35,36,37,54]; and (ii) Rad52 stimulates Rad51 in an RPA-independent manner [37,53,55], perhaps by stabilizing the Rad51-ssDNA filament [55].

RPA plays a dual role in HR. Being added after Rad51 to ssDNA substrate, it stimulates DNA strand exchange between long (plasmid size) DNA molecules. Two mechanisms of stimulation were reported. RPA promotes formation of the Rad51-ssDNA filament, an active species of DNA strand exchange [56] by removing DNA secondary structures [57]. RPA also binds the ssDNA strand displaced during DNA strand exchange preventing the reversal of the reaction [57]. However, when RPA binds to ssDNA prior to Rad51, the order of protein addition that likely mimics the in vivo situation, it competes with Rad51 for ssDNA binding inhibiting nucleoprotein filament formation and DNA strand exchange [57]. This inhibitory effect of RPA is alleviated by Rad52 [35,36,37,58,59], which interacts with both RPA [46,60] and Rad51 [36,37,61] acting as a mediator in promoting recruitment of Rad51 to the RPA-coated ssDNA. Interestingly, the mediator activity was demonstrated in vitro for *S. cerevisiae* Rad52, but not for the human ortholog, even though both yeast and human RAD52 physically interact with their Rad51 and RPA counterparts [62,63,64]. In humans, RAD51 loading on RPA-covered ssDNA was shown to be mediated by BRCA2 [38,65]. 

### 3.3. DNA Invasion or Strand Exchange

Although Rad52 bears no structural homology to Rad51, it is able to promote DNA strand exchange between ssDNA and short linear dsDNA or supercoiled plasmid dsDNA, albeit less efficiently than RAD51 [33,34,66]. In contrast to Rad51/RadA/RecA family of recombinases, Rad52 does not hydrolyze or bind ATP or other nucleotide cofactors. Thus, Rad52 adds to the list of structurally unrelated proteins including *Escherichia coli* RecT and eukaryotic Hop2 that promote DNA strand exchange in an ATP-independent manner [67,68,69]. In contrast to Rad51, Rad52 does not form helical filaments with ssDNA. Instead, it forms heptameric rings with a central channel [70]. On ssDNA Rad52 forms nucleoprotein structures that are composed of stacked rings or edge-to-edge rings [66]. Still, Rad52 shares many properties with Rad51/RecA recombinases. For instance, human RAD52, similar to Rad51/RecA, contains two DNA binding sites that are required for DNA strand exchange [33,71]. The primary site responsible for formation of RAD52-ssDNA complexes forms a deep positively charged groove that runs around the outside of the ring [42]. The secondary site is closely aligned with the primary site and is responsible for interaction with dsDNA during DNA strand exchange or with ssDNA during DNA annealing [33]. In addition, similar to Rad51/RecA, human RAD52 unwinds dsDNA [33]. Furthermore, during the search for homology, both Rad51/RecA and human RAD52 form large co-aggregates containing ssDNA and dsDNA [72,73,74]. Important details of the mechanism of RAD52-promoted DNA strand exchange remain to be elucidated, including the forces that drive the reaction forward and cause RAD52 dissociation from the product. 

DNA strand exchange (DNA pairing) activity of RAD52 suggests that it may play a role of an alternative recombinase in vivo, at least in some HR events. Some published data are consistent with this RAD52 activity. For instance, it was reported that in BRCA1- and PALB2-depleted cells, DSB-induced recombination between chromosomally integrated directly repeated sequences of the GFP gene (DR-GFP) was decreased further approximately 10-fold, when RAD52 was knocked down [13]. In addition, RAD52 may promote RAD51-independent break-induced replication (BIR) by catalyzing ssDNA invasion (DNA strand exchange) into duplex DNA [7]. However, currently there is no direct evidence for the role of RAD52 DNA strand exchange activity in vivo.

## 4. Role of the Structural Domains of Rad52

**Figure 2:**
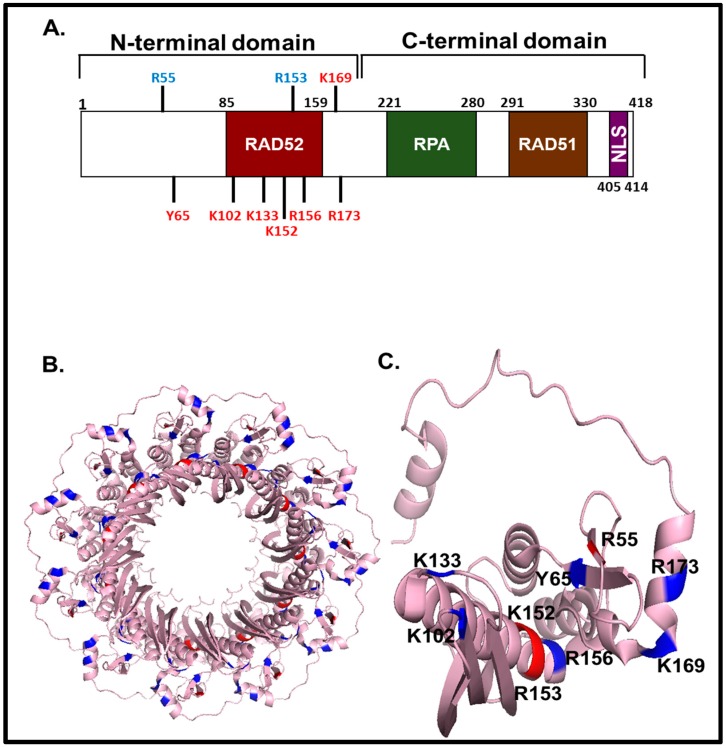
Structure of human RAD52: (**A**) The domain map of human RAD52. The N-terminal domain (NTD) contains the DNA binding region, a self-associating region; the C-terminal domain (CTD) contains RPA and RAD51 interacting regions and a nuclear localization signal. (**B**) The structure of the undecamer ring formed by RAD52 NTD_1–212_(PDB ID:1KN0) [75]. (**C**) The structure of RAD52 NTD_1–212_ monomer. Amino acid residues marked with red bind to both ssDNA and dsDNA; amino acid residues marked with blue bind to ssDNA only. Structures in B and C were prepared using PyMOL Molecular Graphics System, Version 1.2r3pre, Schrödinger, LLC.

Structural studies and mutational analysis provided a physical framework for the DNA strand exchange, ssDNA/ssRNA annealing, and the mediator activity of Rad52 [33,75,76,77]. Rad52, whose size varies in different species from 504 aa (*S. cerevisiae*) to 418 aa (humans), consists of two domains, the N-terminal domain (NTD) and C-terminal domain (CTD), that divide the protein in two roughly equal parts (Figure 2A). 

**Figure 3:**
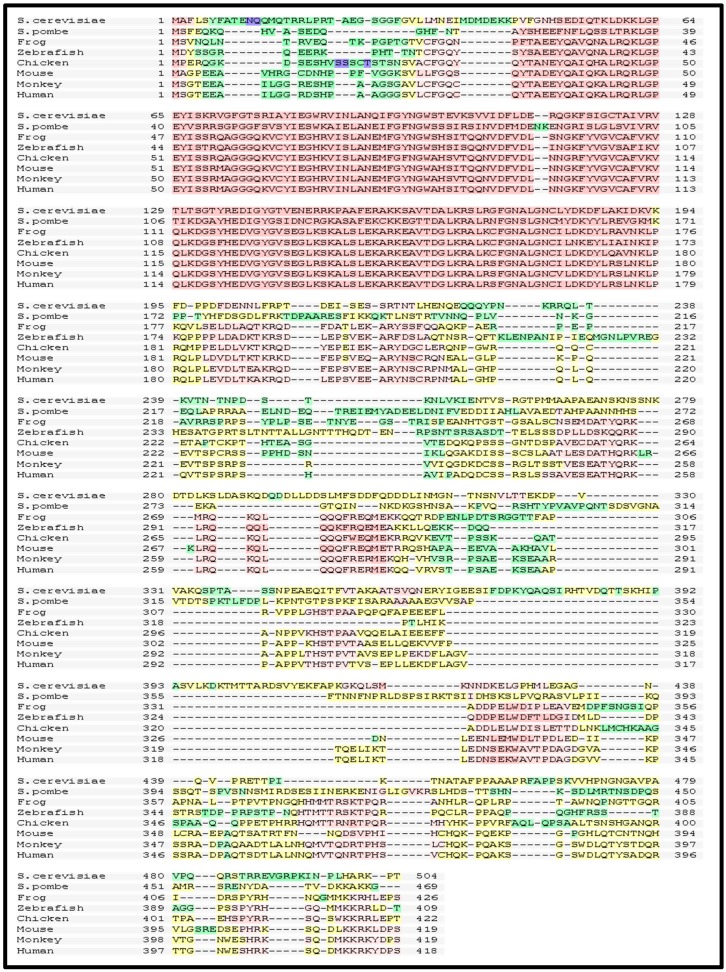
Sequence conservation of RAD52 orthologs. The RAD52 aa sequences from *Saccharomycescerevisiae, Saccharomyces pombe* (RAD22)*,* frog (*Xenopus laevis*), zebrafish (*Danio rerio*), chicken (*Gallus gallus*), mouse (*Mus musculus*), monkey (*Macaca mulatta*), and humans (*Homo sapiens*)were analyzed using multiple sequence alignment program, T-coffee [83]. Pink, yellow, green, and blue colored regions show high, low, very low, and no conservation among sequences, respectively.

The Rad52 NTD is well-conserved among eukaryotes; for instance, the NTDs of *S. cerevisiae* and humans share 42% of identity (Figure 3). In contrast, the CTD is poorly evolutionarily conserved [78,79,80,81].

NTD and CTD play different roles in the Rad52 functions: NTD is involved in binding to ssDNA and dsDNA and Rad52 multimerization (Figure 2) [66,77,82], whereas the CTD is responsible for interaction with RPA [64] and Rad51 [63] (Figure 2A).

### 4.1. DNA Binding

Using a filter binding and mobility shift assays it was shown that both yeast and human RAD52 bind various types of DNA with the following preference: ssDNA > tailed dsDNA >blunt-ended dsDNA [46,84,85]. Binding to supercoiled φX174 dsDNA was about twice as efficient as that to linear dsDNA. 

Using CTD-truncated human RAD52 protein it was shown that the NTD is responsible for DNA binding [66,86]. Structure based alanine scan mutagenesis of RAD52 NTD residues revealed five amino acids, Arg55, Tyr65, Lys152, Arg153 and Arg156, that are directly responsible for DNA binding, with Arg55 and Lys152 being specifically required for ssDNA binding and Tyr65, Arg153, and Arg156 for binding to both ssDNA and dsDNA [75]. Alanine replacements of Phe79 and Tyr81 also resulted in a strong ssDNA binding deficiency [77], however their effect on DNA binding may be indirect [19,77]. More recently, a putative secondary RAD52 DNA binding site was identified that is comprised of Lys102, Lys133, Lys169, and Lys173 [33] (Figure 2B,C). 

In yeast Rad52, but not in human RAD52, a site that binds both ssDNA and dsDNA was identified in the CTD [87]. It was suggested that this site corresponds to the secondary DNA binding site of human RAD52 located in the NTD [44]. In support of this hypothesis, it was demonstrated that the CTD truncation of yeast Rad52 reduces the efficiency of ssDNA annealing, whereas the CTD truncation of human RAD52 has no effect on ssDNA annealing [44]. 

Using fluorimetric method with chemically modified etheno ssDNA (εDNA) the binding site size of yeast Rad52 was estimated to be 10 nt per Rad52 monomer [46]. Using hydroxyl-radical footprinting or FRET assay the binding site size was estimated to be 4 nt per monomer of human RAD52, either full length or the NTD_1–209_ [42,43,88]. The cause for this discrepancy is unknown. Although species-specific features of Rad52 orthologs may play a role, it is possible that the fluorimetric method detects εDNA binding to both the primary and the secondary Rad52 DNA binding sites, whereas other methods visualize only primary site binding. 

### 4.2. RAD52 Multimerization

Electron microscopy studies showed that human RAD52 forms heptameric rings with a large central channel [70,72]. The RAD52 NTD domain retains the ring structure [66,86], but form undecameric (11-mer) rings (Figure 2B) [42,75]. Yeast Rad52 also forms rings, however their detailed structure remains to be determined [46]. In the presence of ssDNA, RAD52 forms filamentous complexes composed of stacked rings or edge-to-edge packed rings [66,89]. RAD52 also forms higher order structures composed of multiple heptameric rings, which is mediated by the presence of self-association region in the CTD (residues 193–418) [86]. Thus, the NTD and CTD have different roles in RAD52 multimerization. NTD is responsible for the formation of a heptameric ring of the full-length RAD52 and undecameric ring in the absence of CTD. The CTD, which does not form ring structures, is responsible for the formation of higher order complexes. Since NTD can promote ssDNA annealing and DNA strand exchange alone, formation of higher order complexes seems to be not essential for these RAD52 activities. However, quantitative studies are needed to determine the effect of the CTD on the rate and extent of these reactions. 

### 4.3. Interactions of RAD52 with RPA, RAD51 and MUS81

Both yeast and human RAD52 interacts with their RPA [46,60,64] and RAD51 [36,37,61,63] counterparts. In yeast, genetic studies demonstrated that deleting the Rad51 and RPA interacting regions of the yeast Rad52 protein partially impairs the recombination activities in vivo [90,91]. In vitro and in vivo studies demonstrated that human RAD52 CTD regions spanning 221–280 aa and 291–330 are essential for interaction with RPA [64] and RAD51 [63], respectively (Figure 2). Furthermore, it was shown that the RQK motif of human RAD52 (residues 261–263) is important for interaction with RPA [43]. Previously, the RQK motif responsible for interaction with RPA was also identified in human DNA annealing helicase SMARCAL1 [92]. RPA is a trimeric protein that consists of RPA70, RPA32 and RPA14 subunits [41,93]. Immunoprecipitation and ELISA-based assays revealed strong interaction of human RAD52 with RPA32, weak interaction with RPA70 and no interaction with RPA14 [64,94]. Using light scattering analysis, it was shown that the binding of RPA trimer to RAD52 CTD (residues 218–418) results in disruption of the higher order RAD52 structures [94]. 

MUS81 is a structure specific endonuclease with a preference for nicked Holliday junctions, D-loops, or three way junctions [95,96]. It was shown in pull-down experiments that purified His-tagged RAD52 interacts with MUS81 in HeLa nuclear extracts [97]. Moreover, RAD52 and MUS81 functionally interact to promote cell viability in checkpoint-deficient cells under replication stress; simultaneous loss of both these proteins leads to cell death that can be rescued by depletion of RAD51. These data indicate a role of RAD52 during repair of stalled replication forks. The link between Mus81 and Rad52 orthologs in repair of damaged replication forks was also reported in *S* *accharomyces pombe* [98,99]. 

### 4.4. Nuclear Localization Signal: Cellular Localization of RAD52 and its Truncated Isomers

The C-terminal domain contains a stretch of eight amino acids (residues 411–418), which is important for RAD52 nuclear localization [100]. Deletion of these eight residues abolished its recruitment to DSBs. In contrast to the yeast *RAD52* gene, which has no introns, the open reading frames of the human *RAD52* are divided into 12 exons. Splice variants of human RAD52 have been isolated from cDNA libraries derived from both brain and testes tissues [101,102]. The RAD52 β, γ and δ isoforms have C-terminal truncations and consist of 226-, 139- and 118-aa residues, respectively. The C-terminal ends of the isoforms comprise unique sequences resulted from alternative splicing. The truncations resulted in complete loss of RPA and RAD51 binding domains and partial loss of self-association domain. Thus, the β, γ and δ isoforms are unable to interact with each other and also with RAD52α, but retain their ssDNA and dsDNA binding activities [101]. The full-length RAD52 protein (RAD52 α) due to the presence of nuclear localization signal is confined to the nucleus. The isoforms localize to both the cytoplasm and nucleus, because being smaller in size they can penetrate the nucleus through the nuclear pore. The functional role of the RAD52 isomers is yet to be determined. Interestingly, in parallel with human RAD52 isoforms, *S. serevisiae* carries the Rad59 protein of 339 aa, which has significant homology with the Rad52 NTD and shares some of the Rad52 in vivo functions and in vitro activities including ssDNA annealing [103,104,105,106]. 

## 5. Regulation of RAD52 in the Cell

### 5.1. Cell Cycle Regulation of RAD52 Protein

In response to DNA damage, RAD52 forms nuclear foci, which are thought to represent the sites of DNA repair [25]. In yeast and mammals, RAD52 foci formation in response to DNA damage is under control of cell cycle; it gradually increases when cells enter S phase, reaches its peak in S phase and then fades out as the cell enters G2 phase [25,107,108]. In contrast, non-homologous end-joining (NHEJ) is the dominant repair pathway in G1 and G2 phases. In yeast, where the process was studied in detail, Rad52 recruitment to DSB sites does not depend on DNA replication per se, as cells that have entered S phase, but have not replicated their DNA, readily form Rad52 foci in response to DNA damage [109]. However, CDK1-cyclin B kinase activity is required for the Rad52 recruitment. The mechanism how CDK1 regulates this process is unknown; CDK1 may act directly on the Rad52 or phosphorylate an upstream factor like RPA. 

### 5.2. Post-Translational Modification of RAD52

Several studies analyzing the role of RAD52 in yeast and higher eukaryotes have revealed post-translational modifications of RAD52, although the role these modifications may vary in different species. In *S. cerevisiae*, Rad52 is constitutively phosphorylated at serine and/or threonine residues through the cell cycle and additional phosphorylation occurs in S phase, but not after gamma irradiation [110]. The phosphorylation was not observed in the Rad52 truncation mutant lacking the CTD, however, the exact phosphorylation sites are yet to be determined. In *S. cerevisiae,* the biological role of Rad52 phosphorylation remains to be determined. In *S. pombe* Rad52 phosporylation was induced under conditions of oxidative stress or in cells deficient in Rad51 or Mus81 [111,112]. Similar, in humans RAD52 is phosphorylated in response to DNA damage at Tyr104 by c-ABL tyrosine kinase in an ATM and DNA-PKcs dependent manner [113]. Phosphorylation led to enhanced RAD52 activities; it stimulated RAD52 foci formation [113] and ssDNA annealing activity [114]. By using a stable phosphotyrosine analog p-Carboxymethyl-L-phenylalanine (pCMF) incorporated into RAD52, Honda et al., examined the mechanism of how phosphorylation by c-ABL kinase affects the activity of RAD52 [114]. They found that incorporation of pCMF (RAD52^Y104pCMF^) increases a RAD52 binding preference for ssDNA compared to dsDNA, and stimulates its ssDNA annealing activity.

In *S. cerevisiae, S. pombe,* and humans, RAD52 is also modified by post translational addition of small ubiquitin-like modifier (SUMO). In *S. cerevisiae* Rad52 sumoylation is induced by DNA damage and triggered by formation of MRE11-Rad50-Mrx2 complexes with DSBs both in meiotic and mitotic cells [115]. The residues involved in sumoylation, Lys10, Lys11, and Lys220, lie in a relatively less conserved region of NTD, suggesting that modifications may not perturb the essential functions of this domain [115]. Although sumoylation-defective Rad52 is proficient in HR, sumolyation stimulates its function by protecting Rad52 against proteasome-mediated degradation [115,116]. Sumoylation of Rad52 also stimulates its interaction with Rad51, whereas cell cycle dependent kinase Cdc48 disrupts SUMO-Rad52-Rad51 complexes [117]. Furthermore, sumoylation of Rad52 is also responsible for exclusion of Rad52 foci from the nucleolus, which thereby suppresses deleterious ribosomal DNA recombination [118]. 

Sumoylation of human RAD52 was also observed in HEK293T cells [115]. Using SUMO-expressing *E. coli,* sumoylation of the RAD52 was detected and the site of sumoylation was mapped at the putative nuclear localization region [100,119]. These data indicate a potential role of sumoylation in the nuclear transport of RAD52. Recently, it was reported that PTEN, an important tumor suppressor, physically interacts with RAD52 in response to DNA damage and is involved in regulation of RAD52 sumolyation in the nucleus [120]. 

## 6. RAD52 in RNA-directed DNA Repair 

Storici et al. demonstrated that synthetic RNA oligonucleotides can act as a template in DSB repair in yeast [121]. In human cells, using an I-Sce endonuclease induced DSB repair system it was also found that DSBs can be repaired using template RNA oligonucleotides [122]. However it was not known whether or not RNA can be used as a template directly, without reverse transcription into DNA. 

Recent work by Keskin et al. demonstrated using reverse-transcription defective yeast strain and endogenous RNA transcripts that RNA can be directly used as a template for DSB repair, without a reverse transcription step [20]. However, this process could only be efficient in the absence of RNases H1/2 that disrupt the RNA-DNA hybrids, an intermediate in RNA-dependent DSB repair. In these experiments, it was also demonstrated that Rad52 plays an important role in RNA-templated DSB repair; RNA transcript-dependent repair was inhibited in Rad52-deficient cells. In parallel, it was shown that in vitro both yeast and human RAD52 promotes annealing between homologous ssDNA and ssRNA, a likely step of RNA-directed DSB repair [20]. The data from human cells that show an RNA-dependent localization of RAD52 at sites of DSBs are consistent with the role of RAD52 in RNA-dependent DSB repair also in humans [123]. Thus, RNA can potentially mediate DSB repair by serving as a template in both yeast and mammals and RAD52 plays a role in this process.

## 7. RAD52 as a Therapeutic Target

New findings that RAD52 is essential for cell viability in BRCA1-, PALB2- and BRCA2- and RAD51 paralog-deficient cells, but not in normal cells, suggested that RAD52 may represent an attractive therapeutic target for killing hereditary breast cancer and ovarian cancer cells. 

The Skorski’s group targeted RAD52 in BRCA-deficient cancer cells using a small peptide aptamer [19]. They designed a 13-aa peptide containing RAD52 sequence surrounding Phe79, which is thought to prevent RAD52 from ssDNA binding by disrupting the assembly of the RAD52 ring structure. It was shown that the aptamer caused synthetic lethality in those selected leukemia patient cells that had a low expression level of BRCA1 or RAD51C. The lethality in these cells was reverted by expression of ectopic BRCA1 or RAD51C indicating specific targeting of RAD52 in human cells. It was also shown that the aptamer enhanced the effect of conventional therapy of leukemia cells with ABL1 tyrosine kinase inhibitors, like imatinib, or cytotoxic agents. 

Small molecule inhibitors were also developed to target RAD52 using different approaches [16,17,18]. Using molecular docking, Sullivan et al. performed a virtual computer screening of libraries of 140,952 FDA approved drugs and drug-like compounds in a search for potential inhibitors of DNA binding by RAD52 [18]. The screening in combination with gel-retardation assay yielded nine small molecule inhibitors of RAD52. One of these compounds, adenosine 5’-monophosphate (A5MP), selectively inhibited the growth of BRCA1-deficient HCC1937 breast carcinoma cells (adenosine was added to the cells and phosphorylated intracellular), even though that RAD52 does not have a nucleotide binding site. 5-Aminoimidazole-4-carboxamide ribonucleotide (AICAR) 5’ phosphate (ZMP), a compound that mimics A5MP, also disrupted RAD52-ssDNA interactions. AICAR showed biological activity in human cells by selective inhibition of growth of BRCA1-deficient HCC1937 breast carcinoma cells, BRCA1-deficient BCR-ABL1-positive leukemia cells, and BRCA2-deficient Capan-1 pancreatic adenocarcinoma cells. AICAR also inhibited formation of RAD52 foci induced in response to cisplatin treatment. 

High throughput screening (HTS) was used by two groups to identify RAD52 inhibitors [16,17]. The Pomerantz’s group used fluorescence polarization to screen libraries of 19,584 drug-like and pharmacologically active compounds for inhibitors of RAD52 binding to ssDNA [16]. The identified compounds were further analyzed for their ability to selectively inhibit SSA in human cells using a GFP reporter system [124]. As a result, a single RAD52 inhibitor, 6-OH-dopa, was identified. Further analyses demonstrated that 6-OH-dopa inhibits RAD52 ssDNA binding by disrupting formation of RAD52 rings and superstructures in vitro. The authors demonstrated the biological effect of the inhibitor in mammalian cells. 6-OH-dopa inhibited RAD52 foci formation in response to DNA damage in murine hematopoietic cells deficient in BRCA1 and selectively killed BRCA1 and BRCA2-deficient human cancer cells.

Our lab in collaboration with the Broad Institute screened libraries of 372,903 compounds including Broad’s diversity-oriented synthesis (DOS) library for inhibitors of RAD52 ssDNA annealing and DNA strand exchange activities [17]. Overall, as a result of the HTS and several confirmatory and selectivity assays, 14 specific inhibitors of RAD52 were identified. Five of these compounds representing 3 different chemotypes selectively inhibited growth of BRCA1-, BRCA2-deficient human cancer cells and BRCA1-deficient (with low expression level) primary cells from leukemia patients. Two of these compounds with the strongest inhibitory effect were studied further. Using Surface Plasmon Resonance (SPR) they were shown to directly interact with RAD52. In cells, they inhibited RAD52 foci formation induced by cisplatin, but not RAD51 foci formation, indicating specific targeting of RAD52 in the cell. Finally, one of the compounds selectively inhibited the RAD52-dependent SSA in human cells. Further work is needed to demonstrate the efficacy of RAD52 inhibitors in killing BRCA1- and BRCA2-deficient cancer cells in vivo. 

While this paper was under review, another study involving development of RAD52 small molecule inhibitors was published by the Spies’s group [125]. Using a FRET-based assay, they screened a library of 2320 drug and drug-like synthetic and natural products for their ability to inhibit RAD52-ssDNA binding and ssDNA annealing. Two inhibitors were selected (“1” and “6”) and their physical interaction with RAD52 was demonstrated. Molecular docking predicted binding of these compounds to the ssDNA binding groove of RAD52 ring. Based on the structure of the proposed RAD52-inhibitor complex an additional RAD52 inhibitor was identified b*y* in silico screening. In cells, it was shown that the inhibitors act additively with depletion of BRCA2 and MUS81 indicating specificity of RAD52 inhibition.

## 8. Conclusions

RAD52 is an evolutionarily conserved protein. In yeast, it plays a major role in all types of recombination events. In contrast, in mammals, the RAD52 knockouts do not show significant deficiency in HR or DSB repair. Recent discoveries, which demonstrated an essential role of RAD52 for viability in BRCA-deficient cells and a novel role of RAD52 in RNA-dependent DSB repair, have an invigorating effect on the recombination field and strongly stimulated research on the functions of RAD52 in mammalian cells. They also prompted development of RAD52 inhibitors, which could lead to novel cancer therapies against hereditary breast cancer and ovarian cancer and other types of cancers in which RAD52 is essential for cell viability due to various deficiencies in HR.
